# Analysis of Single Nucleotide Polymorphisms of Liver X Receptor Alpha (LXR-α) Gene in Diabetic Kidney Disease

**DOI:** 10.7759/cureus.71981

**Published:** 2024-10-21

**Authors:** Deepa Sinnur, R.S. Bulagouda, Sandeep Patil, Santosh Patil

**Affiliations:** 1 Genetics, Bijapur Lingayat District Educational (BLDE) Shri B.M. Patil Medical College Hospital and Research Centre, Vijayapura, IND; 2 Anatomy, Bijapur Lingayat District Educational (BLDE) Shri B.M. Patil Medical College Hospital and Research Centre, Vijayapura, IND; 3 General Medicine, Bijapur Lingayat District Educational (BLDE) Shri B.M. Patil Medical College Hospital and Research Centre, Vijayapura, IND; 4 Urology, Bijapur Lingayat District Educational (BLDE) Shri B.M. Patil Medical College Hospital and Research Centre, Vijayapura, IND

**Keywords:** diabetic kidney disease, lipid metabolism, lxr-α gene, single nuclear polymorphism, type 2 diabetes

## Abstract

Introduction

Genetic factors significantly contribute to the progression of diabetic kidney disease (DKD), which is one of the world's most common causes of chronic kidney disease (CKD). Inflammation, glucose homeostasis, and lipid metabolism are all significantly influenced by the liver X receptor alpha (LXR-α) gene. Single nucleotide polymorphisms in the liver X receptor alpha gene may result in the altered function of the gene, making the individual susceptible to DKD. The purpose of this article is to examine the frequency of expression of single nucleotide polymorphisms (SNPs) in exon 7 of the LXR-α gene in people with type 2 diabetes (T2DM) and DKD. Gaining an understanding of these genetic differences could improve risk assessment and therapy approaches while providing fresh perspectives on the molecular mechanisms underlying DKD.

Aims and objectives

To identify specific SNPs in the LXR-α gene in T2DM and DKD patients.

Methods

The study was conducted between May 2022 and May 2023 and it involved 120 T2DM and 120 DKD patients. All the clinical history of the study participants were recorded. Blood samples were collected from the participants and genomic DNA was isolated and was followed by polymerase chain reaction (PCR) using exon 7 specified primers of LXR-α gene. To find mutations in the LXR-α gene, the PCR products were subsequently analyzed using the capillary-based Sanger sequencing technique.

Results

A total of seven SNPs were detected in T2DM patients, and 20 SNPs were detected in DKD patients. SNP rs7120118 was found with the highest frequency of 100 (83.33%) in T2DM patients and 80 (66.66%) in DKD patients. SNP rs1956299169 was found to have the second highest frequency of 60 (50%) among T2DM patients and SNP rs1252683195 was found to have a frequency of 20 (16.7%). Other common SNPs were rs1956299169, with 40 (33.33%) in DKD and 60 (50%) in T2DM, and rs12574081, with 20 (16.71%) in T2DM and 10 (8.33%) in T2DM.

Conclusion

The study detects the highest and the most common SNP rs7120118, which may be associated with T2DM and DKD. Thereby, it helps to conduct further association studies with larger populations and to diagnose the disease.

## Introduction

Liver X receptor alpha (LXR α) mutations are important when it comes to Type 2 Diabetes Mellitus (T2DM) and Diabetic Kidney Disease (DKD). Various mutations may affect the pathophysiology of various disorders as well as lipid metabolism and liver health. Mutations in the LXRα gene may cause altered functionality, such as loss of function, or noticeable adverse effects. Specifically, harmful mutations in the LXRα ligand-binding domain can result in abnormal lipid metabolism and are associated with decreased triglyceride levels and increased high-density lipoproteins (HDL) cholesterol. Despite these changes in lipid levels, these mutations were also linked to increased serum liver enzymes, indicating possible hepatotoxicity [1]. LXR alpha plays a complex role in type 2 diabetes because it affects several metabolic pathways, including the metabolism of fatty acids and cholesterol. LXR agonists have been demonstrated in studies to dramatically lower blood glucose levels and improve glucose tolerance in mice with diabetes. These findings highlight the relationship between LXR activity and glucose homeostasis regulation, highlighting the latter's potential as a therapeutic target. LXR deficiency has been linked to higher DKD severity. This correlation is most likely caused by the role of LXR in regulating cholesterol efflux and lowering the accumulation of cholesterol in renal cells, a defining feature of DKD. Furthermore, rs7120118, one of the LXR-alpha gene polymorphisms, has been connected to an increased risk of developing DKD, indicating that genetic variations may have a function in the development of renal illness in people with diabetes [2].

LXRα is essential for maintaining homeostasis of fats and cholesterol. Localization within the cell, the transactivation process, protein-protein interaction, and binding of ligands were all impacted by mutations in the LXRα interface residues. While LXRα, L414R, and R415A continued to bind to 25-hydroxycholesterol, they did not bind to T-0901317. Charge reversal at the interface selectively affects LXRα dimerization, and every LXRα mutation showed reduced ligand-dependent luciferase activity levels [3]. Nuclear receptors are known as liver X receptors that repress transcription, which can be raised using N-CoR cotransfection of cells. Corepressors are drawn to native LXR target genes and LXR agonists release N-CoR from those that promote them. LXRs control the expression of genes in reaction to oxysterols and are important for maintaining cholesterol homeostasis [4]

Hyperglycemia and dyslipidemia are common metabolic diseases associated with T2DM. The nuclear receptors farnesoid X-receptor (FXR) and LXR are important in the pathophysiology of T2D. Current studies investigate their physiological and pathological functions in controlling the metabolism of lipids, glucose, and bile acid, and they might be beneficial for therapy to treat type 2 diabetes [5]. LXRs are present in two different forms, α and β, and have been linked to key metabolic processes such as the generation of bile acid and the regulation of lipid and glucose levels. Although recent research indicates that LXRs may have significant modulatory roles in adipocytes and adipose tissue, it is still unknown what the LXRs' clinical function in adipose tissue is [6]. LXRs α and β are important kidney-related proteins that facilitate cholesterol outflow and suppress inflammatory responses. According to a study, whereas LXR agonism may slow down the progression of renal disease in diabetes, LXR deficiency within a mouse prototype for the disease may hasten the collection of intraglomerular cholesterol. The study discovered that renal lipid buildup and the albumin: creatinine ratio increased in those with LXR impairment, indicating a novel treatment target for diabetic nephropathy [7].

The LXR-α variant rs7120118 is especially important when considering India, where the number of cases of DKD and T2DM is alarmingly rising. By exploring the genetic tendency within the Indian community, more can be learned about the intricate connections between genetic predisposition, lifestyle factors, and environmental influences that result in various metabolic disorders. Developing these links could be beneficial for specific public health initiatives aimed at reducing the effects of T2DM and DKD in India. Further genetic studies and functional validations are required to ascertain the causal relationships between LXR alpha variations and T2DM and DKD. Larger cohort studies are needed to verify findings and look into the underlying processes by which rs7120118 influences metabolic health. Such research could play a significant role in the development of precision medicine strategies for treating and preventing various disorders in the Indian context.

## Materials and methods

The study enrolled patients from diabetic and kidney hospitals from six different districts of north Karnataka. The study was carried out at the Center for Advanced Medical Research Centre (CAMR) between May 2022 and May 2023. An Institutional Ethical Certificate (approval number: BLDE (DU)/IEC/608/2022-2023) dated 28/5/2022 was acquired from the Bijapur Lingayat District Educational (BLDE) (Deemed to be University) Institutional Ethical Committee.

Sample size calculation

The sample size for the current investigation was established using a reference study by Liu et al. [2]. In order to achieve a power of 99% for detecting a difference in proportions between two groups at a two-sided p-value of 0.05, the study would need a sample size of 120 cases (DKD) and 120 cases (T2DM), assuming equal group sizes. This is because the anticipated proportion of TT among T2DM and DKD is expected to be 62.4% and 49.5%, respectively. Formula used is

n=(zα + zβ)^2^ 2 p*q/MD^2^

Where Z= Z statistic at a level of significance, MD= Anticipated difference between two proportions, p= Common proportion, and q= 100-p.

Selection of patients

Informed consent was obtained from study participants prior to the collection of blood samples. The study included 120 DKD and 120 T2DM patients in total.

Inclusion criteria

The DKD criteria were established by the National Kidney Foundation Kidney Disease Outcomes Quality Initiative (NKFQDOQI) guidelines, and T2DM was defined by the Indian Council of Medical Research (ICMR) guidelines. Patients diagnosed with a minimum of five years of T2DM and no kidney issues participated in the T2DM group. Individuals with a minimum of 15 years of T2DM with an estimated Glomerular Filtration Rate (eGFR) rate below 60 ml/min/1.73 m^2^ are included in the DKD group.

Exclusion criteria

Individuals with nephrotic syndrome, AKI (Acute Kidney Disease), Lupus nephritis, UTI (Urinary Tract Infection), and pregnant women were excluded from the study.

Collection of blood samples

The phlebotomists of the respective hospitals collected 1 ml of blood sample in the morning after fasting for six to eight hours in ethylene diamine tetraacetic acid potassium (K2 EDTA) vacutainers. The tubes were stored at 4⁰C until the next process began. DNA extraction, polymerase chain reaction (PCR), and sequencing were conducted for more investigation.

DNA extraction

Deoxyribonucleic acid (DNA) isolation was carried out using a G-sure kit (GCC Biotech, G4625, Bengaluru, India), which employs a silica membrane-based purification approach. The quantity and quality of DNA were evaluated using the Infinite 200 Pro-Tecan plate reader (Tecan, Mannedorf, Switzerland). The DNA samples were stored at -20º until the next use. The primers for intronic and exonic junctions of exon 7 were designed using a bioinformatics software tool called Primer3 (www.primer3.ut.ee). The designed primers were synthesized by the Oligo Synthesizer Laboratory (Europhins, India). The primer specifics are noted in Table 1.

**Table 1:** Primer description LXR: Liver X-Receptor

^Name of Primers^	^Sequence of primer^	^Annealing temperature^
^LXR-α F^	5′-TGTGCTGCCTGGATGTATTG-3′	^61º^
^LXR-α R^	5′-CTCTGAGGGTCTGCTGATGC-3′	

Genotyping for polymorphism detection

Polymerase chain reaction (PCR) was utilized to confirm genotyping using the TaqMan single nucleotide polymorphism (SNP) Genotyping Assay (Applied Biosystems, Waltham, MA, USA) and the ABI PRISM 7500 Sequence Detection System (Applied Biosystems). In order to amplify LXR-α via PCR, a total amount of 25 μL reaction mixture was employed, containing 12.5 μL 2× Taq Master Mix (Takara, Shiga, Japan), 1 μL DNA, and 3 μL of each probe (Applied Biosystems). The PCR was conducted under 40 cycles of 92°C for 15 seconds and 61°C for 60 seconds, thereafter, a 10-minute duration of incubation at 95°C. 1% agarose gel electrophoresis was used to validate the PCR results.

After validation, forward and reverse primers were used for each sample to run all PCR products through automated DNA sequencing (ABI_3500xl). Sequence Analysis Software (ABI, Applied Biosystems) was used to assess the sequence quality of the electropherograms produced by automated DNA sequencing. Variant Reporter Software (ABI) was used to achieve sequence alignment.

## Results

There were 120 T2DM and 120 DKD patients in our study. Among them 64 (53.3%) were females and 56(46.7%) males in T2DM group, 62(51.7%) were females and 58(48.3%) males DKD group. Their frequency is tabulated in Table 2.

**Table 2:** Distribution of gender T2DM: type 2 diabetes, DKD: diabetic kidney disease

Sl. No.	Gender	T2DM	DKD
1	Females	64 (53.3%)	62 (51.7%)
2	Males	56 (46.7%)	58 (48.3%)

Out of 120 patients with T2DM, we identified seven SNPs. The most prevalent SNP was rs7120118 in 100 (83.33%) individuals with a nucleotide change from Thymine to Cytosine (T>C). 60 (50%) patients had rs1956299169. The remaining SNPs rs12574081, rs1252683195, rs2135676896, rs144395212 and rs2135679200 were present in 20 (16.7%) individuals (Table 3 ).

**Table 3:** List of SNPs of LXR-α in DKD patients (n=120) SNPs: single nucleotide polymorphism, A: adenine, T: thymine, G: guanine, C: cytosine

S.No	SNPs	Frequency	Alleles	Function Consequence	Position
1	rs7120118	100 (83.33%)	T>C	Intronic	47264739
2	rs1956299169	60 (50.00%)	G>A	Intronic	47264901
3	rs12574081	20 (16.7%)	G>A	Intronic	47264863
4	rs1252683195	20 (16.7%)	G>C	Intronic	47264550
5	rs2135676896	20 (16.7%)	G>A	Intronic	47264633
6	rs144395212	20 (16.7%)	T>C	Intronic	47264478
7	rs2135679200	20 (16.7%)	G>A	Intronic	47264898

Similarly, DKD patients showed 20 SNPs. The SNP with the highest frequency was rs7120118 in 80 (66.66%) patients. We also noticed 50 (41.66%) patients with rs44395212, 40 (33.33%) patients with rs1956299169, rs1956268546, rs1956271448, 30 (25.0%) patients with rs1305836016 and rs565665900, 20 (16.66%) with rs887939560, 10 (8.33%) with rs1956272016, rs1956258816, rs12574081, rs2135676896, rs1331368382, rs760640121, rs1956289012 and rs1230130883. We compared the SNPs of both groups and found that all the SNPs of T2DM are repeated in DKD individuals. These SNPs serve as common genetic risk factors for the development of diabetic kidney disease in individuals with type 2 diabetes. The remaining SNPs found only in DKD indicate that they may be responsible for other disorders (Table 4).

**Table 4:** List of SNPs of LXR-α in DKD patients (n=120) SNPs: single  nucleotide polymorphism, A: adenine, T: thymine, G: guanine, C: cytosine

S.No.	SNPs	Frequency	Alleles	Function Consequence	Position
1	rs7120118	80 (66.66%)	T>C	Intronic	47264739
3	rs1956272016	10 (8.33%)	T>A	Intronic	47264603
4	rs1305836016	30 (25.0%)	C>T	Intronic	47264792
5	rs144395212	50 (41.66%)	T>C	Intronic	47264478
6	rs1956258816	10 (8.33%)	G>T	Intronic	47264481
7	rs1956299169	40 (33.33%)	G>A	Intronic	47264901
8	rs1956268546	40 (33.33%)	G>A	Intronic	47264554
9	rs1956271448	40 (33.33%)	C>T	Intronic	47264593
10	rs2135679200	30 (25%)	G>A	Intronic	47264898
11	rs551108611	40 (33.33%)	A>G	Intronic	45934593
12	rs565665900	30 (25%)	G>A	Intronic	47264887
13	rs887939560	20 (16.66%)	A>G	Intronic	47264846
14	rs12574081	10 (8.33%)	G>A	Intronic	47264863
15	rs2135676896	10 (8.33%)	G>A	Intronic	47264633
16	rs1331368382	10 (8.33%)	A>T	Intronic	47264788
17	rs760640121	10 (8.33%)	C>T	Intronic	47264809
18	rs1956289012	10 (8.33%)	C>G	Intronic	47264814
19	rs1230130883	10 (8.33%)	A>G	Intronic	47264551
20	rs889572240	1 (8.33%)	C>T	Intronic	47264728

## Discussion

In our study, exon 7 of LXR-α predominantly shows mutations in the intronic region. SNP rs7120118 is found to be common in both T2DM and DKD groups. Its frequency is highest as compared with other SNPs. Therefore, this research indicates that SNP rs7120118 is strongly connected to T2DM and DKD individuals. These SNPs are involved in the alteration of lipid metabolism, leading to a heightened risk for other metabolic disorders. When we analyzed the SNPs of the two groups, we discovered that every SNP discovered in T2DM was also present in DKD patients. For those with type 2 diabetes, these SNPs act as shared genetic risk factors for the onset of diabetic kidney disease.

Our study is supported by research conducted by Liu et al. in Chinese Han population and it showed that LXR-α rs7120118 C>T polymorphism was associated with diabetic kidney disease susceptibility. The study discovered that, in both dominant and recessive models, rs7120118 was connected to a higher risk of DKD [2]. The other study looked into the relationship between metabolic syndrome (Met S) and polymorphisms in LXR-α rs12221497 G>A and adrenergic receptor beta-3 (ADRB3) rs4994 T>C in Pakistanis. The study discovered that the CC genotype was linked to an increased possibility of Met S, while the Met S risk was shown to be higher in individuals with the GG genotype. The findings suggest these SNPs may increase susceptibility to obesity-related traits [8]. Another study conducted by Zhou et al. found a genetic association between LXR α gene variation and congenital heart diseases (CHD) in the Han population, providing insights into CHD development and prevention mechanisms [9].

Research conducted by Robitaille et al. analyzed the effects of variations in the LXR-α gene on the plasma lipid profile [10]. It found that high plasma total cholesterol and low-density lipoproteins cholesterol concentrations (LDL-C) were more prevalent in carriers of the -115A, -840A, and -1830C alleles. The genotypes c.-840C>A and LXR-α-115G>A explained 2.1% and 1.8% of the variation in total cholesterol and LDL levels, respectively, while the interaction term explained 2·8% (P = 0·005) and 2·9% (P = 0·002), respectively. Higher cholesterol levels in -115A carriers were linked to increased cholesterol intake.similar findings were noted for c.-840C>A and c.-1830T>C. The findings indicated that the plasma lipid profile is modulated by the interaction between cholesterol consumption and LXR-α variations [10].

A study conducted by Rooki et al. investigated the role that genetic polymorphisms play in LXRs to the possibility of metabolic syndrome (MetS) and associated features [11]. Two SNPs that are shared by nuclear receptor subfamily 1, group H, member 3(NR1H3), and NR1H2 were genotyped in MetS patients and controls. Logistic regression analyses showed no significant interactions associated with MetS prevalence. On the other hand, controls had a higher frequency of LXRα haplotype AC, which was linked to a noteworthy protective effect against MetS. The study found that in the Iranian population, these variations are not likely to be associated with MetS and related features [11].

Current research emphasizes the significance of cholesterol buildup in DKD kidneys as a risk factor for renal damage and lipid metabolism disorders. LXRs, as key regulators of lipid metabolism, maintain lipid balance and prevent atherosclerosis [12-16]. Downregulation of LXR-α exacerbates renal lipid deposition and kidney damage, according to a prior work by Suto et al., although The reason why LXR agonists work so well is restricted by substantial adverse consequences [17]. Grzegorzewska et al. discovered that individuals with atherogenic dyslipidemia, having the rs7120118 allele T, had lower levels of the disease compared to those without atherogenic dyslipidemia [18].

One of the main characteristics of diabetes is lipid dysmetabolism, which includes imbalances in sphingolipids, fatty acid oxidation, elevated lipid absorption, and decreased cholesterol metabolism. This results in the generation of reactive oxygen species, Cell death, inflammation, and oxidative stress in DKD. For therapeutic objectives, management of renal lipid dysmetabolism is essential.

The advantage of our research is few studies have been done on the LXR-α gene, despite the fact that it is expressed in the proximal tubules, glomeruli, and renal cortex cells of the kidney, and that its expression maintains the lipid and cholesterol homeostasis in the kidney cells. SNP identification in DKD and diabetes patients enables the creation of individualized treatment regimens. The identified SNPs suggest a hereditary sensitivity to problems in DKD patients and may function as a biomarker for estimating the risk of developing diabetes and DKD.

Study limitations

One of the limitations of our study is the very small population size, which could lower the study's statistical power and make its conclusions less applicable. Additionally, we did not detect any exonic SNPs, which could limit our capacity to identify changes directly altering protein function. Non-exonic SNPs, including those found in regulatory areas, might, nevertheless, be very important in the emergence of disease. To investigate the whole range of SNPs that may be involved in the disease, more research on the remaining exons is required, preferably including larger cohorts and more thorough genetic investigations.

## Conclusions

With a focus on the implications for the Indian population, our study showed a higher frequency of LXR-α rs7120118 in both T2DM and DKD. The investigation of genetic variables of the LXR-α gene highlights the significance of this gene in lipid metabolism and the ensuing risk for DKD. With the use of this genetic technique, high-risk individuals with diabetes mellitus who require closer observation to stop or delay the development of DKD may be identified. More research is necessary to learn further about the biological process of the mutated variant and the association of the variant with DKD.
